# A Single‐Chain Light‐Activatable Transcriptional Reporter for Fluorescently Tagging Mammalian Cells In Vitro

**DOI:** 10.1002/cbic.202500957

**Published:** 2026-04-13

**Authors:** Ola Bartolik, Wenjing Wang

**Affiliations:** ^1^ Life Sciences Institute University of Michigan Ann Arbor Michigan USA; ^2^ Neuroscience Graduate Program University of Michigan Ann Arbor Michigan USA; ^3^ Department of Chemistry University of Michigan Ann Arbor Michigan USA

**Keywords:** AsLOV2, optogenetics, single-chain, transcriptional reporter

## Abstract

Optogenetic tools have revolutionized the control of gene expression with high spatial and temporal resolution. Here we present a Single‐chain Light‐Activatable Transcriptional Reporter (SLATR), a system capable of fluorescently tagging target cells with minutes of white light stimulation. In its inactive, or dark state, a transcriptional factor is cytosolically bound, preventing nuclear translocation. White light irradiation triggers its release through the protease cleavage of a site that is sterically caged by the circularly permuted *Avena sativa* LOV2 (cpAsLOV2) domain. We discovered that cpAsLOV2 cages the cleavage site more efficiently than AsLOV2, achieving low background in the SLATR design. We demonstrate that SLATR exhibits a signal‐to‐background ratio between 3.4 and 36 and achieves reporter activation within 60 min of light stimulation. Furthermore, SLATR outperforms the only other single‐chain light‐activatable transcriptional reporter, LAUNCHER, with faster kinetics, greater light sensitivity, and markedly lower background under identical stimulation conditions. Our single‐chain light‐activatable transcriptional system expands the optogenetic toolkit though providing a simpler system for regulating gene expression with precise spatiotemporal control.

## Graphical abstract

Single‐Chain Light‐Activated Transcriptional Reporter (SLATR) is the first single‐component reporter system to use a constitutively active Tobacco Etch Virus protease (TEVp) on the same chain as the Tobacco Etch Virus cleavage site (TEVcs) with light‐dependent transcriptional readout. Utilizing a circularly permutated AsLOV2 domain (cpAsLOV2) within the SLATR design decreased background signal and could be activated with 1 h of white light stimulation.

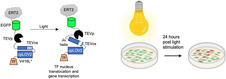

## 1 Introduction

Genetically encoded light‐sensitive (optogenetic) tools have advanced the way researchers investigate and manipulate biological systems by enabling precise control of cellular events with high spatial and temporal resolution. By fusing photoreceptive protein domains to functional effectors, these tools can regulate signaling pathways, protein–protein interactions, and gene expression in response to specific wavelengths of light [1, 2, 3, 4]. Optogenetic approaches circumvent many of the limitations of pharmacological and electrical stimulation, including off‐target effects, slow kinetics, and lack of cell‐type specificity [5, 6, 7]. The diversity of available photoreceptors, ranging from plant‐derived LOV (light–oxygen–voltage) domains to phytochromes and cryptochromes, has facilitated the development of systems that can be tuned for activation or inhibition, reversible switching, genetic editing, and multiplexed control using blue or red light stimulation [3, 8, 9, 10].

Light‐activated transcriptional reporters offer many advantages, such as reporter gene expression amplification and enhanced spatiotemporal control through light irradiation. Several of these light‐activated transcriptional reporters operate through multicomponent designs. For example, photoactivatable Gal4 transcriptional activators work through blue light‐mediated interactions between cryptochrome 2 (Cry2) and its binding partner CIB1, each tethered to split components of Gal4, to reconstitute an active transcription factor under illumination [11, 12, 13, 14]. Similarly, split‐recombinase systems, such as split‐Cre, utilize the Cry2/CIB pair to reconstitute an active Cre recombinase upon blue light illumination, enabling precise temporal control of gene activation [15, 16, 17]. For these multicomponent systems, their signal‐to‐background ratios (SBR) is highly dependent on the protein expression levels of the two components, making optimization for cell cultures and in vivo applications challenging.

More recent tools extend this framework through incorporating additional light gating domains to improve the SBR. BLITz (Blue‐Light Inducible Transcription) leverages the Cry2/CIB interaction and incorporates an additional blue light caging moiety, *Avena sativa* LOV2 (AsLOV2), to release a membrane‐bound transcriptional factor via light‐induced protease reconstitution and proteolytic cleavage [18]. iTango adopts a similar architecture but couples it to the dopamine receptor DRD2, allowing users to record GPCR activation within a defined time window upon blue light stimulation [18]. The SPARK and FLARE systems use improved AsLOV2 caging and a constitutively active tobacco etch viral protease (TEVp) for faster proteolytic cleavage, leading to higher spatiotemporal resolution of GPCR activation or neuronal activity [19, 20, 21, 22]. While these tools offer powerful strategies for precise gene regulation, they often require two or more components, leading to variability in SBR due to nonstoichiometric expression of individual parts. Moreover, integrating such multicomponent architectures into existing synthetic circuits remains a significant engineering challenge.

Currently only one optically controlled, single‐component proteolytic cleavable system, LAUNCHER (Light‐Assisted UNcaging switCH for Endoproteolytic Release), has been developed [23]. This system utilizes a light‐dependent split TEV protease design that incorporates the blue light‐sensitive iLID domain, alongside an iLID‐caged protease cleavage site. However, a minimum of 2 hours of blue light stimulation is required for LAUNCHER activation.

Here we report the design of SLATR (Single‐chain Light Activated Transcriptional Reporter), a single‐chain photoswitchable transcriptional reporter that is activated within an hour of white light stimulation. SLATR utilizes a circularly permuted AsLOV2 (cpAsLOV2) and enables light‐sensitive release of a transcription factor, which then drives the transcription of a user‐defined reporter gene. This reporter gene can fluorescently mark cells, temporally drive protein expression, or be used for further interrogations of labeled cells. We optimized the SBR of SLATR through screening various cpAsLOV2 domains, different TEV cleavage sites (TEVcs), and linker lengths. We demonstrated its sensitivity through proof‐of‐concept light‐inducible transgene expression experiments and benchmarked SLATR's improved sensitivity against LAUNCHER. This highly sensitive photocleavable transcriptional readout provides a generalized method for light‐induced manipulations in cell cultures.

## 2 Results

### 2.1 Prototype Design of a Single‐Chain Photocleavable Switch

To generate a single‐chain light‐dependent transcriptional reporter, we utilized the well‐established light‐regulated tobacco etch virus protease (TEVp) cleavage site (TEVcs) system [24, 25]. Instead of having the TEVp and TEVcs on separate protein chains, we envisioned that combining them into a single protein chain will improve the reporter robustness compared to currently available multicomponent systems. To minimize background proteolysis in the dark state, we used truncated TEVp (1–219, S219V) and low‐affinity TEVcs variants and tested several versions of AsLOV2 domains to cage the TEVcs [26]. To improve light‐dependent uncaging efficiency, we used AsLOV2 variants with the slow reset mutation V416L [27]. In HEK293T cells, we used the Gal4 transcription factor and UAS‐mCherry reporter gene system, which has worked robustly in other transcriptional systems [19, 21]. We utilized ERT2 to restrict SLATR to the cytosol and increase the effective number of the SLATR construct expressed, as opposed to restricting the single‐chain construct to the cellular membrane with limited space. We transduced HEK293T cells with lentiviral vectors coexpressing SLATR and a UAS‐mCherry reporter gene. To evaluate light dependency, we quantified mCherry reporter‐positive cells (activated cell count) in the light and dark conditions and calculated their SBR, defined as the mean number of activated cells in light divided by that in dark.

The caging of the TEVcs in SLATR was not sufficient with the AsLOV2 domain alone, showing high background signal in the dark condition (Figure S1). We then tried double caging the TEVcs with the previously evolved LOV domain, hLOV, caging the N‐terminal portion and cphLOV, and caging the C‐terminal portion of TEVcs (Figure S1) [28]. The double‐caged system with the TEVcs P1 methionine mutation achieved light dependance with SBR of 5.5, but exhibited some background signal (Figure S1). This double‐caged light‐dependent transcriptional system could potentially be further engineered to improve the SBRs in the future.

**FIGURE 1:**
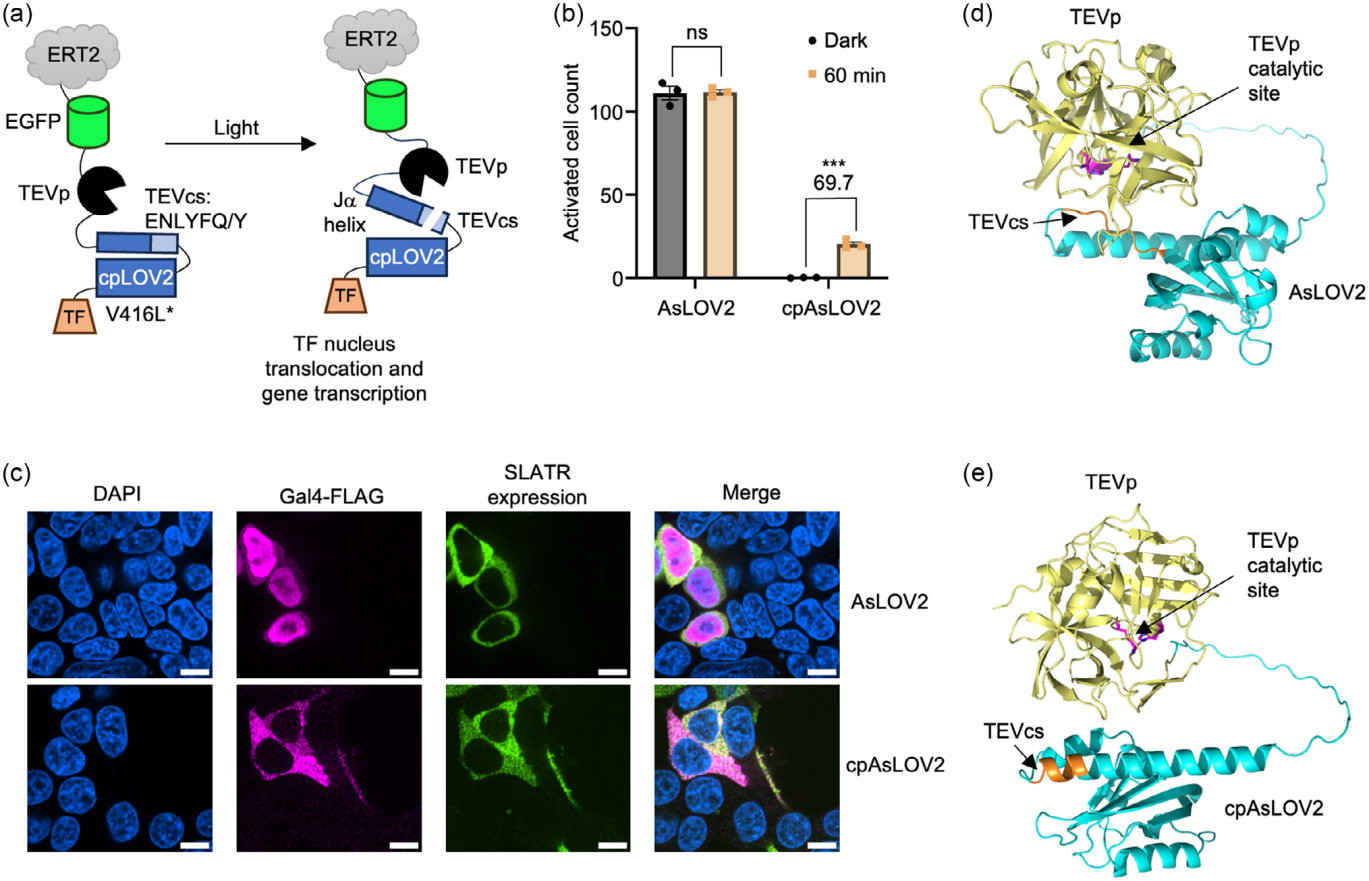
SLATR design utilizing cpAsLOV2. (a) Schematic of SLATR design utilizing cpAsLOV2 with the V416L slow reset mutation. Under light irradiation, cpAsLOV2 uncages the TEVcs allowing for proteolytic cleavage and release of transcriptional factor to activate expression of reporter gene. (b) Testing of SLATR with AsLOV2 domain versus cpAsLOV2 domain in HEK293T cells. Quantification of mCherry positive cells between the dark state and 60 min of white light stimulation at 50% duty cycle (1 min on/1 min off). (c) Confocal imaging at 60× of Gal4‐FLAG localization in dark conditions for SLATR designs incorporating AsLOV2 and cpAsLOV2. DAPI, nuclear staining. (d) Alphafold 3 prediction of TEVp and AsLOV or (e) TEVp and cpAsLOV2 as designed in SLATR, arrows point to TEVcs and TEVp catalytic site. Each dot represents the mean mCherry cell count of one technical replicate. SBRs are calculated by dividing the means between dark and light conditions. Error bars, standard error of the mean (SEM). Stars represent significance after unpaired two‐tailed Student's t‐test. *n* = 3 ***p value < 0.001; ns; no significant difference. Scale bars, 10 µm.

We next examined the cpAsLOV2 domain, which was engineered with the TEVcs inserted at the junction connecting the original C‐ and N‐terminal portions of the AsLOV2 domain [29]. This specific design embeds the TEVcs between the core LOV domain of AsLOV2 and the Jα helix (Figure 1a), rendering the TEVcs less accessible. Incorporating this version of cpAsLOV2 domain with the TEVcs embedded resulted in a significant decrease in dark background cleavage, improving the SBR (Figure 1a,b).

We investigated the localization of Gal4 in the dark state for SLATR incorporating AsLOV2 caging the N‐terminal portion of TEVcs or cpAsLOV2 with TEVcs embedded. Through immunostaining of dark state conditions, we found that Gal4 was localized in the nucleus of cells expressing SLATR with our original AsLOV2 design, indicating light independent cleavage, but remained cytosolic in cells expressing the cpAsLOV2 SLATR design (Figure 1c). These results are consistent with the robust caging observed with cpAsLOV2 in the dark state and the high background signal seen for the SLATR system with AsLOV2. Furthermore, Alphafold 3 predicted the TEVcs to be more accessible as a loop drawn closer to the TEVp active site with AsLOV2, whereas the TEVcs in cpAsLOV2 forms an alpha helix structure. (Figure S1, Figure 1d,e). This configuration of the TEVcs in the alpha helix structure may be due to the TEVcs interfacing more hydrophobic residues of the cpAsLOV2 core domain, resulting in higher caging in the dark state. Whereas in the AsLOV2 configuration the TEVcs are orientated further away from the AsLOV2 core domain, likely resulting in less stability and inefficient caging. We have decided to move forward by optimizing SLATR with cpAsLOV2 for higher SBRs.

### 2.2 Optimizing SLATR

**FIGURE 2:**
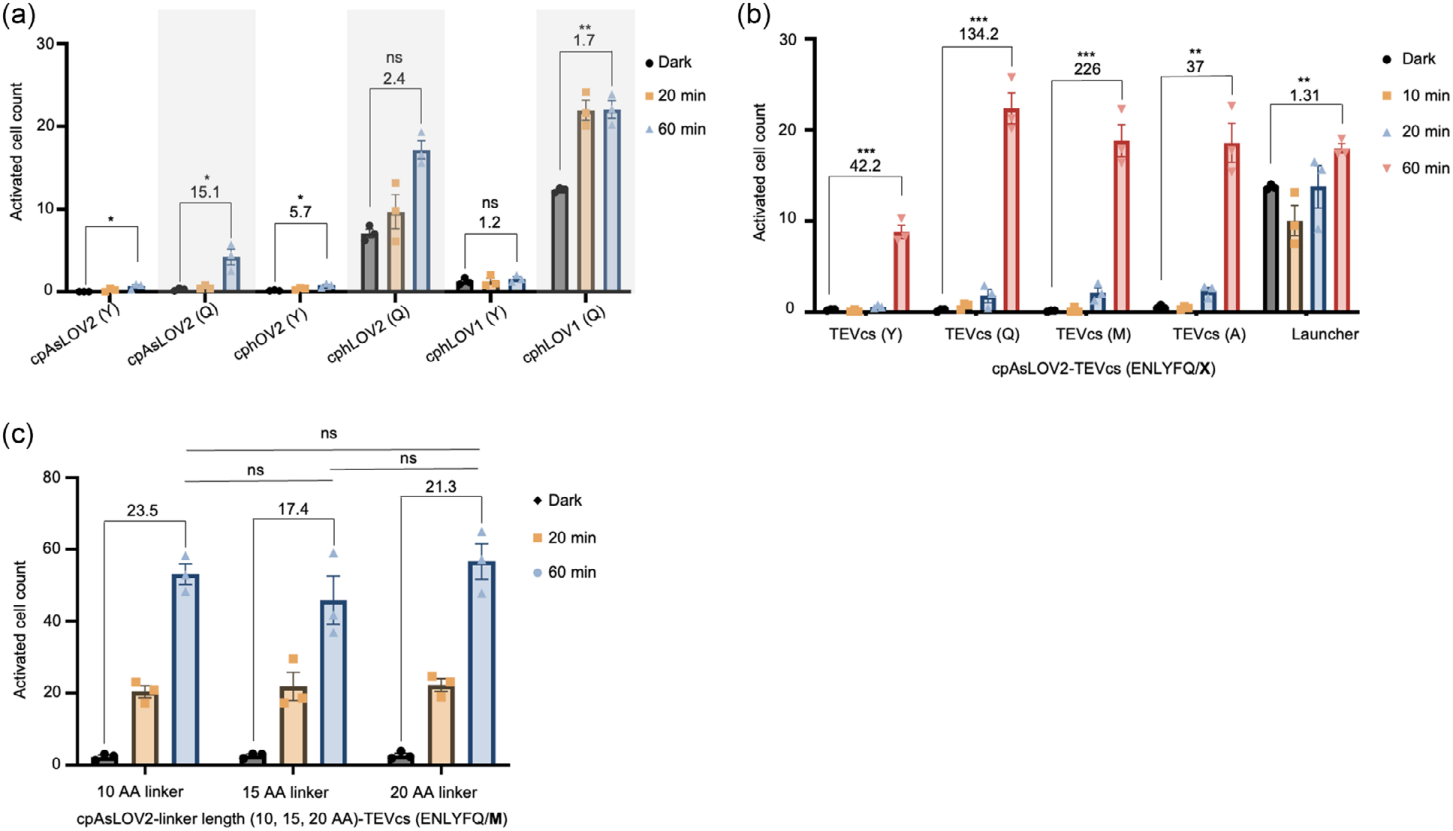
Optimization of SLATR. (a) Screening of different cpAsLOV2 domains and TEVcs in SLATR design. No SBR is provided for cpAsLOV2 (Y) as the dark state had 0 activated cells. (b) Screening of TEVcs with P’1 mutations that impact catalytic cleavage rate. (c) Screening of varying linker lengths between TEVp and cpAsLOV2. Each dot represents the mean mCherry cell count of one technical replicate. SBRs are calculated by dividing the means between dark and light conditions. Error bars, SEM. Stars represent significance after unpaired two‐tailed Student's t‐test and one‐way ANOVA in panel c. *n* = 3 ****p value; <0.0001; ***p value; <0.001; **p value; <0.01: ns; no significant difference.

We further optimized the SLATR system incorporating cpAsLOV2 with TEVcs embedded by screening different circularly permuted versions of previously evolved LOV domains, namely hLOV1 and hLOV2, with slow cleaving and fast cleaving P1’ mutations tyrosine (Y) and glutamine (Q) respectively [19]. We found that SLATR with cpAsLOV2 retained low background accumulation regardless of TEVcs used, whereas SLATR with cphLOV1 and cphLOV2 exhibited higher dark state background with TEVcs of ENLYFQ | Q (Figure 2a). Therefore, we continued the SLATR system with cpAsLOV2.

We next compared the SLATR system, incorporating cpAsLOV2 caging four TEVcs with tyrosine (Y), glutamine (Q), methionine (M), and alanine (A) in the P1’ position, with the LAUNCHER system adapted with UAS‐Gal4. We continued optimizing SLATR with TEVcs with M in the P1’ position as it led to the highest SBR of 226 among the four TEVcs tested (Figure 2b). Notably, the LAUNCHER system adapted with UAS‐Gal4 exhibited much higher background signal than SLATR (Figure 2b). The high background signal of LAUNCHER may be due to differences in the transcriptional reporter used to initially validate LAUNCHER, which was the tTA/Tre system, and here cells were transduced with lentivirus as opposed to transfection used for LAUNCHER validation.

Next, we tested varying the GS linker lengths between the TEVp and the cpAsLOV2 domain from 10 to 20 amino acids. We found that the linker lengths did not significantly impact SLATR activation within the 60‐minute light stimulation groups, so we kept the original 10 amino acid linker (Figure 2c).

We also investigated the impact of the Jα helix length on the caging and light‐dependent uncaging of the TEVcs. Surprisingly, both partially and fully truncating the Jα helix had no impact on the caging of the TEVcs (Figure S2). Follow‐up experiments were done to investigate the light gating of TEVcs in the original AsLOV2 domain. We hypothesized that embedding the TEVcs deeper into the Jα helix of AsLOV2 domain would provide tighter caging as seen in cpAsLOV2 with TEVcs embedded. Placing the TEVcs between the AsLOV2 domain and the Jα helix did not display light dependance, due to insufficient caging resulting in high background signal in the dark state (Figure S2). In contrast to the tight caging observed in cpAsLOV2, this finding suggests that the positioning of the TEVcs within cpAsLOV2 is crucial. The positioning of TEVcs in cpAsLOV likely induces TEVcs helix formation and subsequent interaction with the core LOV domain, resulting in improved caging efficiency. Consequently, cpAsLOV2 caging efficiency is impacted by mutations in the core LOV domain as seen by the change of light dependance in cphLOV SLATR versions.

**FIGURE 3:**
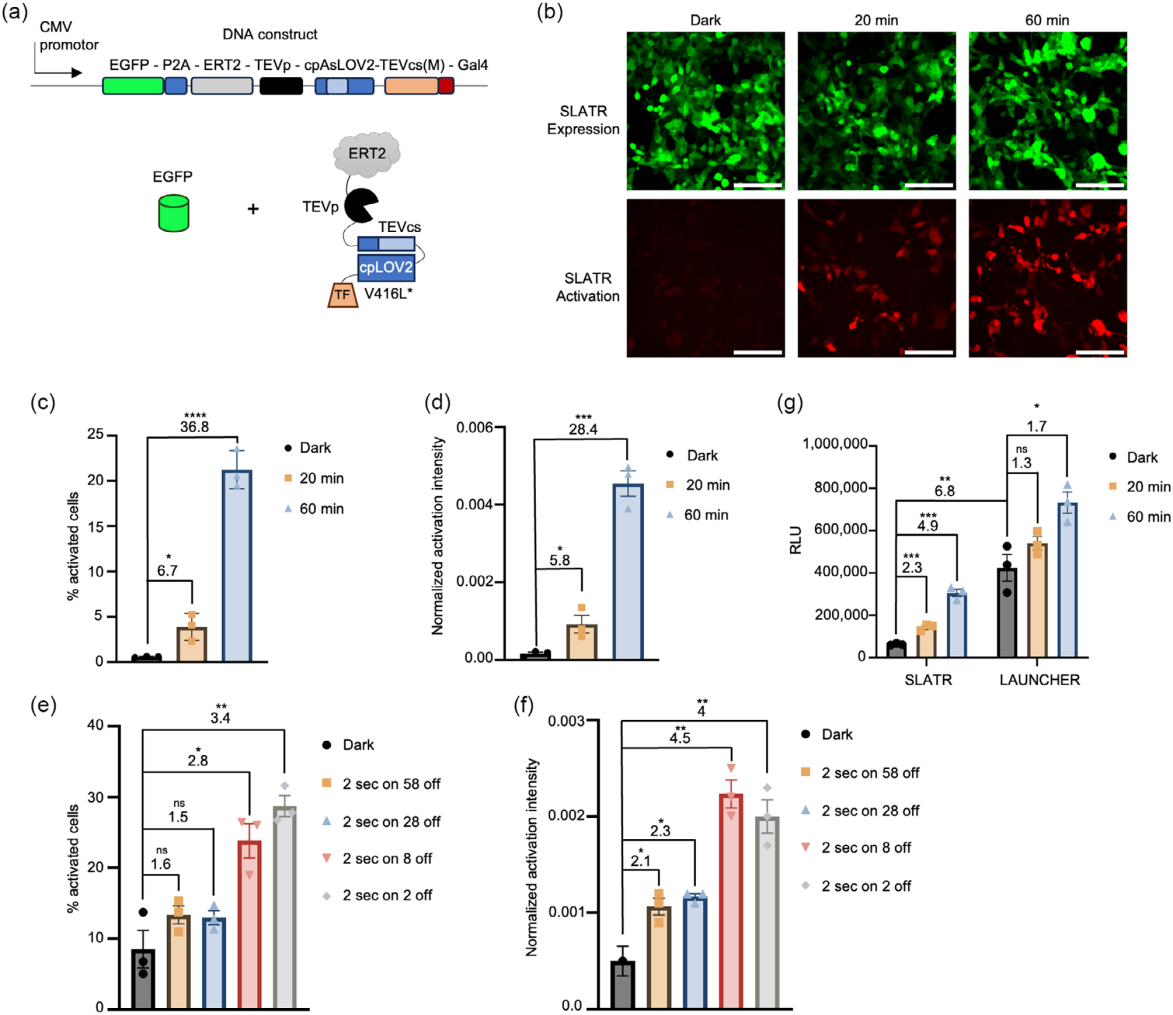
Characterization of SLATR. (a) Schematic of separating SLATR expression signal through P2A‐induced ribosomal skipping between EGFP and SLATR sequence. (b) Representative images of SLATR expression and activation signal between white light stimulation conditions at 50% duty cycle (1 min on/1 min off) in HEK293T cells. (c) Quantification of activated SLATR cells normalized to expression SLATR cells across white light stimulation durations. (d) Quantification of sum SLATR activation intensity normalized to sum SLATR expression intensity across white light stimulation durations. (e) Quantification of SLATR cell activation normalized to cell expression count over differing white light pulse durations over a 60‐minute stimulation duration. (f) Quantification of SLATR intensity activation normalized to SLATR expression intensity with differing white light pulse durations over a 60‐minute stimulation duration. Each dot represents the mean quantification of cell counts or fluorescence intensity of 10 images per well. (g) Quantification of luminescent signal from UAS‐Luciferase reporter across white light stimulation durations. While light stimulation was set at 50% duty cycle (1 min on/1 min off). Each dot represents the luminescence signal from each technical replicate. *n* = 3. SBRs are calculated by dividing the means between dark and light conditions. Error bars, standard error of the mean. Stars represent significance after unpaired two‐tailed Student's t‐test. *n* = 3 ****p value < 0.0001; ***p value < 0.001; **p value < 0.01; *p value < 0.05; ns; no significant difference. Scale bar, 100 µm.

To ensure Gal4 release was specifically mediated by TEVp proteolytic cleavage rather than an artifact of light stimulation, we constructed two control SLATR variants. For a negative control, we removed the TEVp, thereby preventing TEVcs cleavage even upon light irradiation. For the positive control, we constructed SLATR without cpAsLOV caging the TEVcs. This construct was expected to have high autocleavage of the TEVcs and high activation signal even in the dark. Surprisingly, we found the expression level of the positive control SLATR to be lower than the negative control SLATR, while exhibiting high mCherry activation signal (Figure S3). This finding suggests that after proteolytic cleavage of the TEVcs, the single‐chain structure becomes highly unstable and is prone to degradation, thus reducing EGFP signal marking expression. Therefore, we are unable to perform ratiometric analysis of SLATR with EGFP in the single chain as the EGFP fluorescence intensity is negatively impacted by protease cleavage. To ensure the stability of EGFP, which indicates SLATR construct protein expression level, we redesigned SLATR to coexpress EGFP separately utilizing a P2A self‐cleaving peptide (Figure 3a).

We then moved forward with characterizing the optimized EGFP‐P2A version of SLATR, which consists of cpAsLOV2 with the V416L slow reset mutation, methionine in the P1’ position of the TEVcs, and 10 AA linker between TEVp and cpAsLOV2 domain.

### 2.3 Characterizing SLATR

We characterized our final SLATR design with varying pulses and durations of white light stimulation and quantified activation through ratiometric cell count and ratiometric intensity analysis. We utilized these two metrics to demonstrate that not only did overall sum activation intensity increased, but that the number of activated cells also increased with increased light stimulation. SLATR is not restricted to a specific analysis due to similar SBR ratios for both analyses and users may choose either analysis for their specific experimental needs.

To characterize SLATR we first examined SLATR activation by varying white light durations of 20 min and 60 min with 50% duty cycle of 1 min on/1 min off. We found that SLATR requires at least sixty minutes of light stimulation to activate at least 20% of SLATR expressing cells (Figure 3b,c). Ratiometric analysis of SLATR cell count and intensity ratios showed fold changes of 36 and 28 between dark and 1‐hour light stimulation conditions, respectively (Figure 3c,d). Next, we examined SLATR's sensitivity to light by varying the duty cycle in a range from 3.3% to 50%. We expected little difference between the light cycles as the V416L mutation in AsLOV2 was reported to increase the reset time to 71 min; however, this was not observed in with our cpAsLOV2 design in SLATR. In SLATR, the activation increased with increased duty cycles (Figure 3e,f) [27]. These results indicate that the V416L mutation does not elongate the reset time in of cpAsLOV2 in our SLATR design.

We performed one final comparison of SLATR and LAUNCHER utilizing luminescence as the activation readout through the UAS‐firefly luciferase reporter. We found that SLATR has 6.8‐fold lower background signal compared to LAUNCHER (Figure 3g). SLATR was also found to have almost 5‐fold increase between dark and 60 min of white light stimulation (Figure 3g). In contrast, LAUNCHER had a higher dark background signal and only a 1.7‐fold change with 60 min of white light stimulation (Figure 3g). These results support that SLATR offers a more robust single component light activatable transcriptional reporter due to its lower background signal and requires less light activation than LAUNCHER for high SBRs.

## 3 Conclusion

Optogenetic tools have many advantages including rapid kinetics, temporal control, and high sensitivity. SLATR offers these advantages with its simple single‐chain design, low background signal, and ability for user‐defined payloads. SLATR is composed of three functional components: (1) a cytosolic module, (2) a light‐activated switch, and (3) a payload module. We utilized ERT2 to maximize SLATR expression levels in the cytosol, as opposed to being constrained to expressing on the membrane or on an organelle. Our light‐activated switch consists of cpAsLOV2 caging the TEVcs in the dark state and uncaging the site under light conditions, allowing the TEVp to cleave and release the payload. The payload we used was Gal4, which drove expression of mCherry or firefly luciferase; however, it may be used to drive other protein expression readouts to perturb other cellular processes with high spatial and temporal resolution.

SLATR is the first single‐chain light‐activatable transcriptional reporter to use a nonsplit version of TEVp. Many limitations of past transcriptional reporters were due to the need for either split TEVp or a highly evolved AsLOV2 domain to minimize background signal. However, SLATR proves that it is possible to utilize a fully functional TEVp and cage the TEVcs with cpAsLOV2 within a single chain. Furthermore, SLATR is highly sensitive and can be activated within 1 h of white light stimulation, reducing the need for specific blue light equipment to perform experiments. SLATR also outcompetes LAUNCHER with higher light sensitivity and reduced dark state background. This is likely due to the LAUNCHER design relying on caging a split TEV protease whose reconstitution may delay efficient TEVcs cleavage, whereas in SLATR, a constitutively active TEVp allows for rapid cleavage directly upon light stimulation necessitating lower durations of light for effective activation.

While SLATR outperforms LAUNCHER, it still has some limitations. We have observed experimental variations in the SBR of SLATR, mainly due to accumulation of background signal in the dark state. Further optimizations of SLATR will be required to apply it for rigorous experiments in vivo. These limitations could be addressed by directed evolution of cpAsLOV2 for faster cleavage kinetics, allowing for activation with less light stimulation. There have been multiple rounds of mutagenesis of AsLOV2, but so far, none have been reported for cpAsLOV2 [27, 30, 31]. Directed evolution on cpAsLOV2 may also identify variants that further decrease background activity, improving SLATR or other transcriptional integrator tools that utilize AsLOV2. Further optimization to make SLATR more sensitive would allow it to be activated by low energy two‐photon light. This would give SLATR higher spatial resolution and enable it to be coupled to real‐time sensors, tagging cells of interest for further analysis. Here we present a novel single‐chain transcriptional reporter, SLATR, which outperforms existing single‐chain sensors and sensitively provides light‐activated reporter gene expression.

## 4 Materials and Methods

### 4.1 Cloning

Constructs for protein expression in HEK293T/17 cells (RRID: CVCL_1926, ATCC, FISHER, Cat. CRL‐11 268) were cloned into ampicillin‐resistant pLX208 lentiviral vector containing a CMV promotor. Standard cloning procedures such as NEB restriction enzyme digest, PCR amplification with Q5 polymerase, or synthesized gene blocks from TwistBio were ligated through Gibson assembly. Plasmids were transformed into XL1‐blue competent cells using heat shock transformation and plasmids were purified using NucleoSpin Plasmid miniprep kit (Takara).

### 4.2 Structural Prediction Through Alphafold3

Alphafold3 was used to predict cpAsLOV2 and AsLOV2 caging. Amino acid sequences were inputted into the Alphafold3 server and the lowest energy model was chosen to be visualized in Pymol.

### 4.3 HEK293T Cell Culture, Lentivirus Production, and Infection

HEK293T cells with a passage number below 20 were cultured at 37°C under 5% CO_2_ in complete growth media composed of 1:1 DMEM (Dulbecco's Modified Eagle Medium, Gibco): MEM (Modified Eagle Medium, Gibco), 10% FBS (Fetal Bovine Serum, Sigma), 20 mM HEPES (Gibco) and 1% Pen Strep (Gibco).

For lentivirus production for each construct, 1 µg of SLATR viral DNA, 0.9 µg of delta8.9, and 0.1 µg of pVSVG lentiviral helper plasmid were combined with 100 µL of DMEM and thoroughly mixed with 10 µL of PEI MAX. After a 15‐minute incubation at room temperature, 100 µL of complete growth media was added to the DNA–PEI mixture and transferred to a 6‐well plate. Cells were then plated at 70–90% confluency. After incubation at 37°C under 5% CO2 for 36–48 h, the lentivirus supernatant was collected, flash frozen in liquid nitrogen, and stored at –80°C for up to 2 years.

For lentivirus infection in each well of the 48‐well plastic plates, a mixture of 100 µL of SLATR lentivirus and 50 µL of UAS‐reporter gene lentiviruses was well mixed with 200 µL of 50% confluent HEK293T cells. The cell lentivirus mixture was transferred to each well of the 48‐well plate (Corning) and cells were incubated for 36–48 h before stimulation. Plates were covered in aluminum foil to block ambient light sources until light stimulation.

### 4.4 HEK293T Cell Stimulation for Light‐Dependent Protease Cleavage Testing

After lentivirus transduction, HEK293T cells were stimulated with white light from a desk lamp (120V, 60 Hz, 3.5 W) with 1.82 mW/cm^2^ at 473 nm with a working distance of 13.5 cm. The desk lamp illuminates a square area of 24 cm by 24 cm (576 cm^2^). Duty cycle controls of the LED panel were established with Nearpow multifunctional infinite cycle programmable plug‐in digital timer. Cells were then incubated at 37°C for 24–28 h before live‐cell fluorescence imaging. EGFP‐P2A‐SLATR transduced cells were incubated with Hoechst dye (1:1000) (Thermofisher) 10 min before live cell imaging for cell count quantification analysis.

### 4.5 HEK293T Immunostaining for Monitoring Gal4 Trafficking

After lentiviral transduction for 48 h, HEK293T cells were fixed with 4% paraformaldehyde for 15 min and permeabilized with 100% methanol for 5 min at –20°C. Cells were washed with 1× PBS solution twice and then incubated with 1:1000 mouse‐α‐FLAG primary antibody (Sigma, Cat no. F1804) in 1% bovine serum albumin (BSA) for 30 min. Cells were then washed with 1× PBS three times and in 1:1000 mouse‐α−647 (ThermoFisher, Cat no. A‐21 235) in 1% BSA. Cells were washed with 1× PBS three times and then imaged.

### 4.6 Confocal Microscopy

Confocal imaging was performed with a Nikon inverted confocal microscope with a 10× air objective, 20× air objective, 60× oil immersion objective, Yokogawa CUX‐X1 5000 RPM spinning disk confocal head, Ti2‐ND‐P perfect focus system 4, and a 4‐line laser source containing: 405 nm (95 mW), 488 nm (95 mW), 561 nm (60 mW), and 640 nm (70 mW) lasers. The combinations of laser excitation and emission filters were used as follows: EGFP (488 nm excitation; 526/36 emission); mCherry (568 nm excitation; 605/52 emission); Hoechst (405 nm excitation; 435/85 emission); Cy5 (647 nm excitation; 663/738 emission. The acquisition time for all HEK293T cell images was 1 s with 50% laser power intensities, except for 405 nm with an acquisition time of 500 ms and 50% laser power. Images were taken with the 20× objective lens unless otherwise noted. All images were collected using Nikon NIS‐Elements hardware control.

### 4.7 HEK293T Luminescence Assay

For luminescence assays, HEK293T cells were plated in 96‐well opaque white plates. After light stimulation, cells in each well were incubated in complete growth media at 37°C for 24 h. Before luminescence measurement, the plate was cooled to room temperature for 5 min and 60 µL of Bright‐Glo (Promega, Cat no. PRE2620) was then added to each well. Luminescence was immediately measured on a BioTek Cytation 5 cell imaging multimode reader using a 1 s acquisition time and gain at 255.

### 4.8 Quantification and Statistical Analysis

#### 4.8.1 Fluorescence Data Analysis

For HEK293T cell experiments 8–10 fields of view per well were imaged and three technical replicates were performed. Each image was processed and the mean of the images per well was plotted as a single dot, unless otherwise noted. The number of wells processed for each condition is denoted as “n” in the figure legend, unless otherwise noted. NIS‐Elements General Analysis 3 software was used for fluorescence intensity and cell count analysis. For each experiment, the mean intensity for each emission wavelength in an area devoid of cells was used to determine the background for the respective excitation laser against the plate. A threshold was used at twice this level of background intensity for 488 and 405 nm laser emission and threefold this level of background intensity for 568 laser emission to generate a mask of cell areas with real fluorescence for each emission wavelength and the sum intensity and object count were determined. Fluorescent area sizes less than 5 µm were excluded to filter out bright puncta that was not representative of a positive cell. mCherry positive cell counts were determined through object count in NIS‐Elements. For cell counts accounting for SLATR expression, a double mask of Hoechst positive and either 488 nm laser excitation signal or 568 nm laser excitation signal was made to count cells expressing SLATR and cells activated by SLATR, respectively. The cell counts of activated cells and expression cells were then divided by each other to measure total cell activation efficiency. Similarly, sum intensity of 488 nm laser emission and 568 nm laser emission under the threshold mask were divided by each other to measure activation efficiency ratiometrically.

For all comparison experiments, Prism GraphPad software was used to perform unpaired two‐sided Student's *t‐*tests or one‐way ANOVA with comparison of the mean of each condition to each other, to calculate significance and plot data. All images were included in analysis.

SLATR sequence (EGFP, P2A, HA tag, ERT2, MKII, NNES, 10 GS linker, TevP, 10 GS linker, cpAsLOV2 (517–540), TEVcs, cpASLOV2 (1–517), NLS, Gal4, FLAG tag).

## 5 Author Contributions

O.B. and W.W. conceived the reporter designs and experimental designs for HEK293T cell testing. O.B. cloned SLATR constructs, performed HEK293T cell experiments and analyzed the data. O.B. and W.W. wrote the manuscript.

## 6 Supporting Information

Additional supporting information can be found online in the Supporting Information section.

## Funding

This study was supported by NIH (DP2MH132939, R01DA053200), Camille Dreyfus and Henry Foundation (TC‐23−084), Alfred P. Sloan Foundation (FG‐2024‐22156), and National Science Foundation (DGE‐2241144).

## Conflicts of Interest

The authors declare no conflicts of interest.

## Supporting information

Supplementary Material

## Data Availability

The data that support the findings of this study are available from the corresponding author upon reasonable request.
