# Supplementary material for: A Single‐Chain Light‐Activatable Transcriptional Reporter for Fluorescently Tagging Mammalian Cells In Vitro
Source: Chembiochem. 2026 Apr 13;27(7):e202500957. doi: 10.1002/cbic.202500957 (PMC13071867; doi:10.1002/cbic.202500957)
Supplement: Supplementary file 1 — Supplementary Material [file CBIC-27-e202500957-s001.pdf]

Supporting Information

A single-chain light-activatable transcriptional reporter for fluorescently tagging mammalian cells in vitro

Ola Bartolik[1,2], Wenjing Wang[1,2,3]

[1] Life Sciences Institute, University of Michigan, Ann Arbor, MI, USA

[2] Neuroscience Graduate Program, University of Michigan, Ann Arbor, MI, USA

[3] Department of Chemistry, University of Michigan, Ann Arbor, MI, USA.

Corresponding email: wenjwang@umich.edu

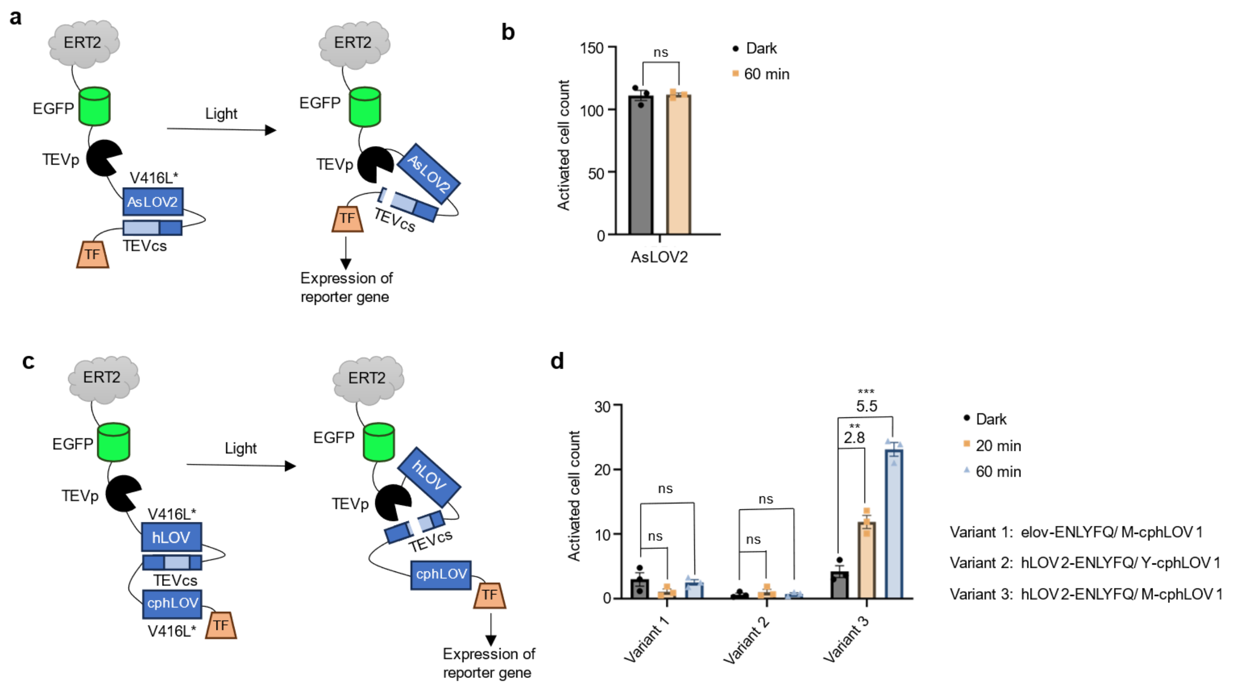

Figure S1. Testing the various designs of SLATR. (a) Schematic of AsLOV2 with slow reset mutation V416L in SLATR design. **(b)** Cell counts of activated cells in AsLOV2 V416L SLATR with TEVcs ENLYFQ/Y. Cells were stimulated with white light at 50% duty cycle (1 minute on/1 minute off). **(c)** Schematic of double-caged SLATR with hLOV2 and cphLOV1. **(d)** Cell counts of activated cells in three versions of double caged SLATR. Variant A uses eLOV and cphLOV1 with the TEVcs of ENLYFQ/M. Variant B uses hLOV2 and cphLOV1 with the TEVcs of ENLYFQ/Y. Variant C uses hLOV2 and cphLOV1 with the TEVcs of ENLYFQ/M. SBRs are calculated by dividing the means between light and dark conditions. Error bars, standard error of the mean. Stars represent significance after unpaired two-tailed Student's t-test. n=3 ***p value <0.001; **p value <0.01; ns no significant difference.

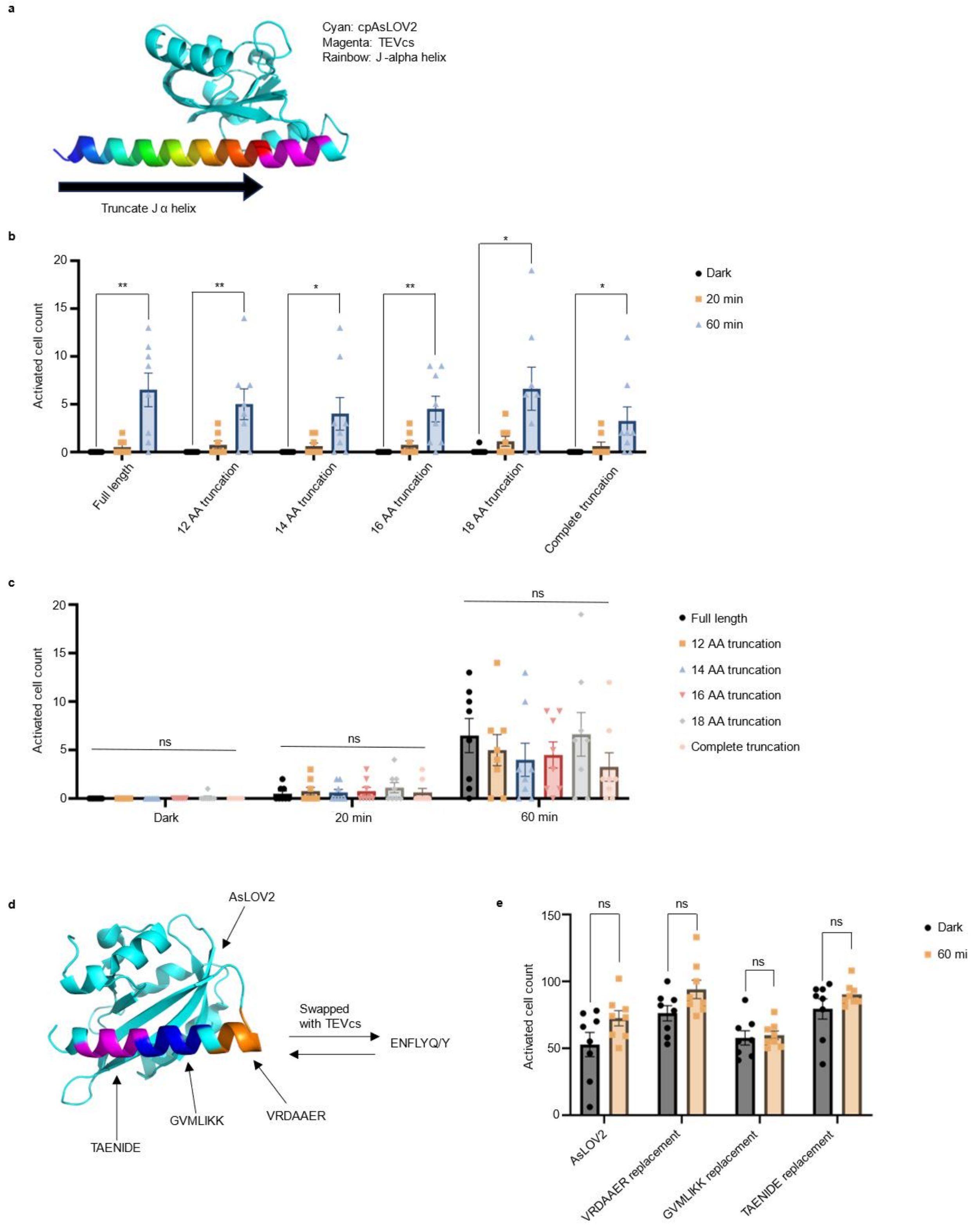

Supplemental figure 2. Investigating the caging of cpAsLOV2. a) Alphafold prediction of cpAsLOV2 with rainbow colored helix displaying the Jα helix and arrow points towards direction of truncation. **b)** mCherry positive cell counts of truncated variants of Jα helix in SLATR system

between dark and light conditions. Each dot represents cell counts in one view of the well. SBR not available due to 0 mCherry positive cells being detected in the dark condition in multiple conditions. **c)** Analysis of truncated variants between light stimulation conditions. One-way ANOVA test was performed comparing the means between each Jα-helix truncation in each light stimulation condition. **d)** Crystal structure of AsLOV2 (2V1A PDB) colored sections highlight helix replacement with TEVcs. Arrows point to amino acids replaced in the Jα helix with TEVcs ENFLYQ/Y. **e)** mCherry positive cell counts of Jα helix replacements in the SLATR system with V416L mutation in AsLOV2. Cells were stimulated with white light pulses at 50% duty cycle (1 minute on/1 minute off for 60 minutes). Each data point represents cell count in one view of the well. n=8. SBRs are calculated by dividing the means between light and dark conditions. Error bars, standard error of the mean. Stars represent significance after un unpaired two-tailed Student’s t-test. n=3 ***p value <0.001; **p value <0.01; *p value <0.05; ns no significant difference.

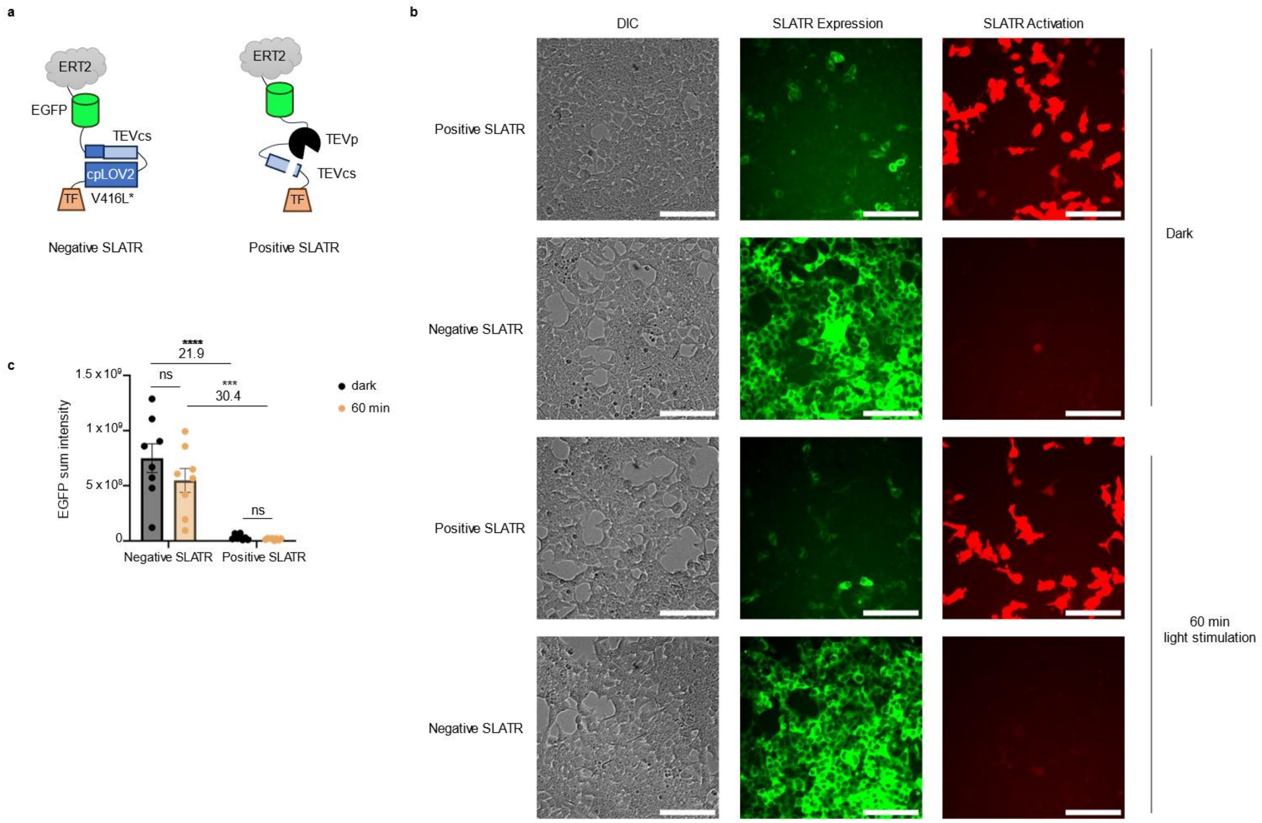

Supplemental figure 3. Loss of SLATR expression signal after proteolytic cleavage of TEVcs. (a) Schematic of negative responding and positive responding SLATR. Negative control SLATR lacks TEVp while positive control SLATR lacks the cpAsLOV2 domain caging the TEVcs. **(b)** Representative images of positive SLATR and negative SLATR in the dark state and with 60 minutes of white light stimulation at 50% duty cycle (1 minute on/1 minute off) **(c)** Quantification of SLATR expression signal between negative responding SLATR and positive responding SLATR. Each dot represents the sum intensity of one field of view. Error bars, standard error of the mean. Stars represent significance after un unpaired two-tailed Student's t-test. n=8 ****p value<0.0001; ***p value <0.001; ns no significant difference. Scale bars, 100 µm.
